# Advanced Strategies for Poly(3-hydroxybutyrate-co-3-hydroxyvalerate) Production: PHA Synthase Homologous Overexpression in the Extremophile *Haloferax mediterranei*

**DOI:** 10.3390/md23040166

**Published:** 2025-04-11

**Authors:** Alexandra Simica, Yolanda Segovia, Alicia Navarro-Sempere, Rosa María Martínez-Espinosa, Carmen Pire

**Affiliations:** 1 Multidisciplinary Institute for Environmental Studies “Ramón Margalef”, University of Alicante, Ap. 99, E-03080 Alicante, Spain; alexandra.simica@ua.es (A.S.); rosa.martinez@ua.es (R.M.M.-E.); 2 Biotechnology Department, Faculty of Science, University of Alicante Ap. 99, E-03080 Alicante, Spain; yolanda.segovia@ua.es (Y.S.); alicia.navarro@ua.es (A.N.-S.); 3 Biochemistry, Molecular Biology, Edaphology and Agrochemistry Department, Faculty of Science, University of Alicante Ap. 99, E-03080 Alicante, Spain

**Keywords:** *Haloferax mediterranei*, PHA, PHBV, PHA synthase, overexpression, haloarchaea

## Abstract

Bioplastics such as poly(3-hydroxybutyrate-co-3-hydroxyvalerate) (PHBV) are promising alternatives to conventional plastics. However, the high production cost limits their industrial application. In this study, PHBV production was optimized in *Haloferax mediterranei* by the homologous overexpression of the key enzyme PHA synthase (PhaEC), resulting in the OE*phaEC* strain. The growth and PHBV production of OE*phaEC* compared with the parental strain (HM26) were evaluated in three culture media with different nitrogen sources (KNO_3_, NH_4_Cl, and casamino acids). The OE*phaEC* strain exhibited a 20% increase in PHBV production and a 40% increase in 3-hydroxyvalerate monomer (3HV) content in a defined medium with nitrate as a nitrogen source, as determined by GC-MS. Moreover, enzyme activity, measured spectrophotometrically, increased from 2.3 to 3.9 U/mg. Soluble and insoluble protein fractions were analysed to assess the overexpression of PHA synthase. Only PhaE was found in the insoluble protein fraction, where PHBV granules accumulate. Transmission electron microscopy (TEM) images confirmed a higher PHBV content in OE*phaEC* compared to the parental strain. These results demonstrate that the homologous overexpression of the key enzyme implicated in PHBV biosynthesis can enhance PHBV content, making its production competitive for industrial applications.

## 1. Introduction

Global plastic pollution is one of the main current concerns due to the extensive use of petroleum-derived plastics (most of them not recyclable) and the accumulation in nature of microplastics and nanoplastics [1,2,3,4]. To mitigate this impact, there is a growing interest in sustainable alternatives, such as biopolymers, showing bioplastic properties derived from plants or microbial biomass [5]. Among these, polyhydroxyalkanoates (PHAs) stand out as a promising solution. PHAs are linear polyesters that accumulate in the cytoplasm of various microorganisms as a source of energy and carbon under conditions of scarcity of essential nutrients and excess carbon [6,7]. Their most attractive properties include complete biodegradability tested in many environments, biocompatibility, sustainability, thermoplasticity, strength, and elasticity [5,6,8].

There are several types of PHAs, of which poly(3-hydroxybutyrate-co-3-hydroxyvalerate) (PHBV) is particularly relevant due to its lower crystallinity and greater elasticity compared to polyhydroxybutyrate (PHB) [9]. These properties make PHBV more similar to conventional plastics and better suited for medical and biotechnological applications, including tissue engineering and wound healing [5,10,11]. The production of PHAs has been documented in over 70 bacterial and archaeal genera inhabiting many ecosystems including oceans, solar salterns, the rhizosphere, and plant surfaces [8,12,13]. Most PHA-producing bacteria are Gram negative, but their application in PHA production is complex due to endotoxin contamination during PHA extraction, elevating final production costs as well as the use of large amounts of organic solvents, which are not environmentally friendly [12,14]. Although Gram-positive bacteria synthesize lower quantities of PHAs, their use lacks commercial interest due to purification challenges, particularly the accumulation of triacylglycerols by some strains [12,15,16]. Another drawback of bacterial PHA production is the need for media sterilization in most cases (except for extremophilic ones), which increases energy consumption. In this context, extremophilic microorganisms, which thrive in different extreme conditions, align well with the terminus “Next-Generation Industrial Biotechnology” (NGIB) and offer promising advantages for PHA production, such as reducing the need for sterilization and enabling more cost-effective processes [17,18].

Hence, special attention is directed towards haloarchaea as PHA synthesis factories [19]. These organisms thrive optimally under high salinity conditions, thus making it possible to avoid sterilization [20,21,22]. Additionally, haloarchaea offer advantages such as cellular lysis using distilled water, facilitating PHA granule recovery, and reducing the downstream processing cost [23].

The haloarchaeon *Haloferax mediterranei* has garnered interest as an excellent candidate for industrial PHA production [24,25,26]. As an extremophile, it accumulates PHAs during growth to protect cellular functions under nutrient starvation [10]. Numerous studies highlight its excellent capacity to utilize waste and convert it into PHAs. Moreover, it has the capacity to synthesize PHBV and poly(3-hydroxybutyrate-co-3-hydroxyvalerate co-4-hydroxybutyrate) (PHBV4HB) instead of PHB [27,28,29]. The production of PHBV by *H. mediterranei* has been studied with multiple substrates such as candy industry waste, rice-based ethanol stillage, olive mill wastewater, hydrolysed rapeseed meal, and ricotta cheese whey, promoting a circular economy and the valorisation of industrial by-products [25,30,31,32,33]. Another advantage is its metabolic flexibility that allows PHBV synthesis through multiple pathways without requiring precursors, such as propionate or valerate, which are often toxic to the cells [10,34,35,36]. These properties confer *H. mediterranei* distinct advantages over other PHA-producing microorganisms.

*H. mediterranei* PHA granules consist of 97.5% PHAs, 2% synthesis, regulation and depolymerization proteins, and small amounts of lipids [37,38,39]. Among the enzymes involved in the synthesis and bound to PHBV granules is polyhydroxyalkanoate synthase (PhaEC), which is responsible for polymerising PHA monomers [10,37,40]. In *H. mediterranei*, PhaEC belongs to the type IIIA group of PHA synthases and consists of two subunits, PhaC (54.8 kDa) and PhaE (20.4 kDa) [41]. PhaC is the catalytic subunit while PhaE is essential for the enzyme’s biological activity, although its specific role remains unclear. Genes encoding both subunits are located in a single operon on the megaplasmid pHM332; their coding regions overlap by four nucleotides, and they are controlled by the same promoter upstream of *phaE* [42].

Despite the benefits mentioned above, the biotechnological industry faces significant challenges, such as high bioprocessing times and energy consumption. Different strategies need to be adopted to achieve efficiency and compete with the chemical industry. The production cost of PHBV currently ranges between USD 1.5 and USD 10/Kg, which is not cost effective compared to conventional plastics, which cost between USD 0.5 and USD 1.4/Kg [1,43]. Additionally, the biotechnological production of PHA by wild-type microbial strains is currently economically unfeasible compared to the industrial chemical synthesis of petroleum-derived plastics. In order to promote the large-scale use of PHA through a competitive process in terms of time and cost and to be commercially viable, a key strategy involves the generation of overproducer strains [44]. Many attempts have been made to increase the production of PHAs using *H. mediterranei* as model organism, but none of them have addressed the homologous overexpression of the key enzymes in charge of the biopolymer production to reach a feasible and profitable production of PHBV.

This study presents, for the first time, the homologous overexpression of the PHA synthase of *H. mediterranei* as a key strategy to overproduce PHAs. The growth and PHBV synthesis of the overexpression strain was monitored in defined culture media using different nitrogen sources (ammonium, nitrate, and casamino acids). The enzymatic activity was compared with that of the parental strain. In addition, PHBV granules from both the overexpression of *phaEC* and parental strains were analysed by TEM. This study aims to enhance the understanding and optimization of *H. mediterranei* as a platform for sustainable bioplastic production

## 2. Results

### 2.1. Construction of the phaEC Overexpression Strain

**Figure 1:**
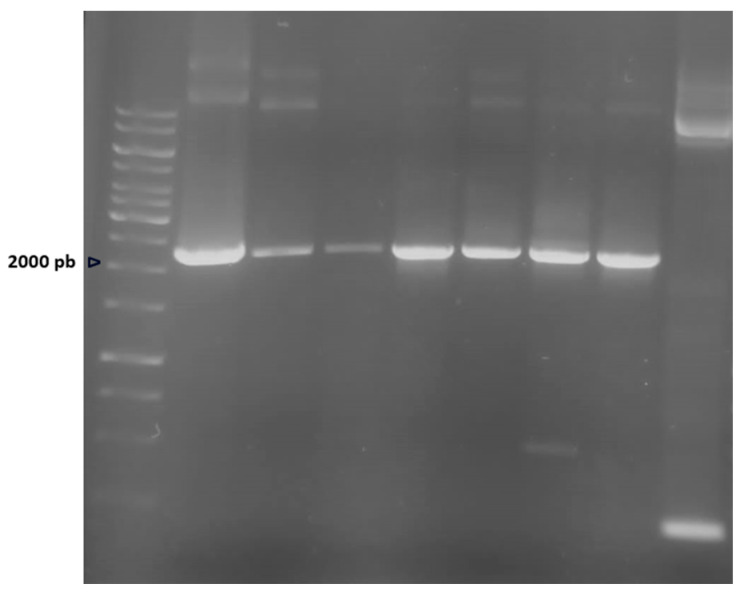
PCR verification of *phaEC* genes in transformed *H. mediterranei* HM26. Lane 1: molecular standard (1 Kb). Lane 2: positive control. Lane 3–8: *phaEC* genes amplified from pTA199*2-phaEC* construction. Lane 9: negative control. The arrow indicates the 2000 pb molecular weight marker.

Construction of the *phaEC* overexpression strain (OE*phaEC*) was carried out as described in the Section 4. For testing transformants of *H. mediterranei* HM26 carrying the pTA1992-*phaEC* construction, amplification of the 2233 pb product by PCR was performed after the extraction of the plasmid. The results showed that all colonies tested carried the recombinant plasmid for *phaEC* overexpression. Figure 1 shows six tested transformants. The template used for the positive control was the pTA1992-*phaEC* plasmid isolated from *E. coli* JM110 and verified by Sanger sequencing, and the negative control was pTA1992 without insert, isolated from *E. coli* DH5α. 

### 2.2. Growth Characterisation of H. mediterranei OEphaEC and Parental (HM26) Strains

**Figure 2:**
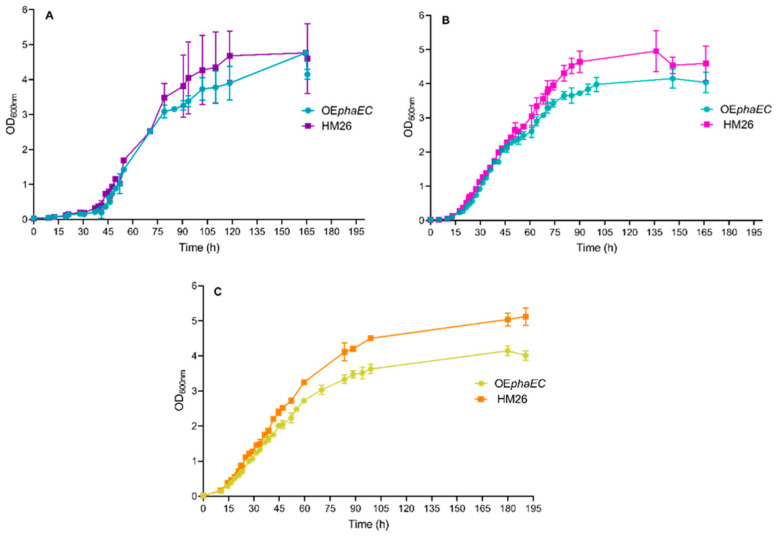
Growth of the *H. mediterranei phaEC* overexpression strain (OE*phaEC*) and parental strain (HM26) under different nitrogen sources. (**A**) 100 mM KNO_3_; (**B**) 10 mM NH_4_Cl; (**C**) 0.5% casamino acids. Data are based on three independent replicates. Plotted values are the mean of triplicate measurements, and error bars represent ± SD.

Characterization of growth kinetics was carried out in three different defined culture media, all of which maintained a constant carbon source of 55.5 mM glucose. To analyse the effect of the nature and concentrations of the nitrogen source, the following three conditions were tested: 100 mM potassium nitrate (KNO_3_), 10 mM ammonium chloride (NH_4_Cl), and 0.5% casamino acids (cas). Figure 2 shows the growth curves of the three cultures.

The lag phase observed from the three-culture media is similar (and even overlapping) in both strains, as well as the beginning of the exponential phase of growth. However, after 70 h of incubation, the parental strain cultures reached higher values of OD_600_ values compared to the overexpression strain, except in the medium with 100 mM KNO_3_, where final OD values were similar in both strains. The final OD_600_ values for the OE*phaEC* strain were 4.76 (±0.07) in nitrate medium, 4.2 (±0.3) in ammonium medium, and 4.14 (±0.11) in casamino acids medium. For the parental strain, the final OD_600_ values were 4.6 (±1.0), 5.0 (±0.6), and 5.12 (±0.19) in nitrate, ammonium, and casamino acids media, respectively.

**Figure 3:**
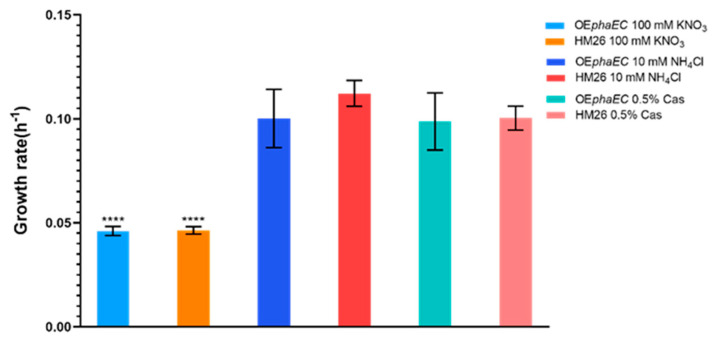
Growth rates (h^−1^) of the PHA synthase overexpression and the parental HM26 strain in the different culture media. *p*-value < 0.0001 (****).

Regarding the growth rates, no significant differences were observed between the two strains in the same medium, but in the different culture media, the lowest growth rate for both strains was observed in the medium containing nitrate (Figure 3), which is the growth rate for the OE*phaEC* strain 0.046 h^−1^ (±0.002) and the parental strain 0.0463 h^−1^ (±0.0018). The values of the growth rates in the medium with ammonium were 0.1001 h^−1^ (±0.014) for the OE*phaEC* strain and 0.1122 h^−1^ (±0.0062) for the parental strain. The growth rates achieved in the medium with 0.5% casamino acids were 0.0990 h^−1^ (±0.014) for the OE*phaEC* strain and 0.100 h^−1^ (±0.006) for the parental strain.

### 2.3. PHBV Quantification in OEphaEC and Parental (HM26) Strains

**Figure 4:**
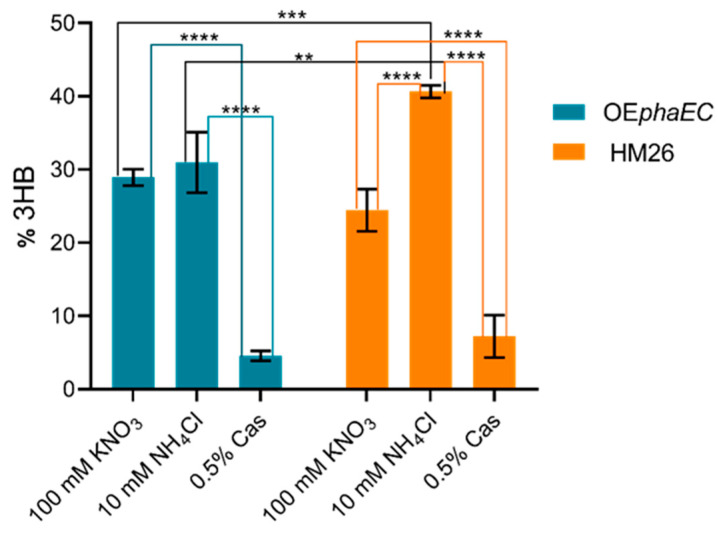
Graphical representation of the mean of 3HB content represented as % by weight of lyophilized biomass of the overexpression strain (OE*phaEC*) and the parental strain (HM26) in media with 100 mM KNO_3_, 10 mM NH_4_Cl, and 0.5% casamino acids (Cas). *p*-value < 0.01 (**); *p*-value < 0.005 (***); *p*-value < 0.0001 (****).

**Figure 5:**
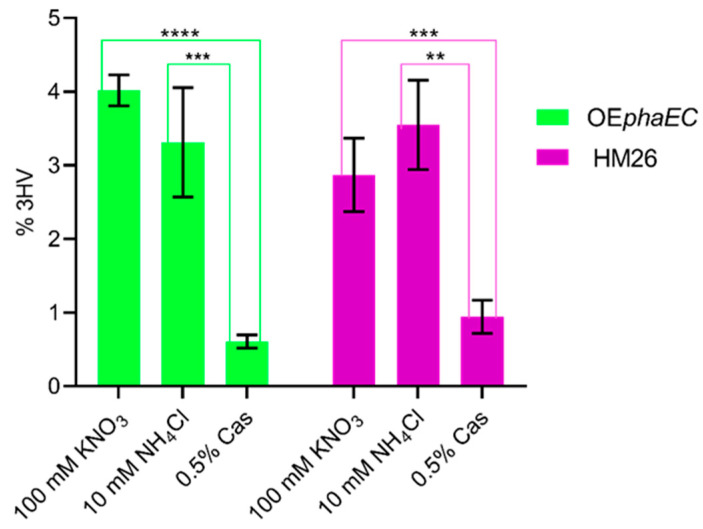
Graphical representation of the mean of 3HV content represented as % by weight of lyophilized biomass of the overexpressing strain (OE*phaEC*) and the parental strain (HM26) in media with 100 mM KNO_3_, 10 mM NH_4_Cl, and 0.5% casamino acids (Cas). *p*-value < 0.01 (**); *p*-value < 0.005 (***); *p*-value < 0.0001 (****).

PHBV quantification was carried out in the three media at the stationary phase. Figure 4 and Figure 5 show the 3-hydroxybutyrate (3HB) and 3-hydroxyvalerate (3HV) content of the two strains in the three distinct culture media, expressed as a percentage of cell dry weight (CDW). These values represent the average of three biological replicates for each condition. Significant differences are indicated by bars of different colours and asterisks, with varying *p*-values. 

In the defined medium with nitrate, the OE*phaEC* strain achieved a 3HB production ((mg 3HB/mg CDW) × 100) of 28.9% (±1.1). In the medium with ammonium, the production reached 31% (±4), while in the medium with casamino acids, it was 4.6% (±0.7). Meanwhile, the parental strain produced 25% (±3) 3HB in the nitrate medium, 40.7% (±0.9) in the ammonium medium, and 7% (±3) in the casamino acids medium.

Regarding 3HV production ((mg 3HV/mg CDW) × 100), the OE*phaEC* strain reached 4.0% (±0.2) in the nitrate medium, 3.3% (±0.7) in the ammonium medium, and 0.61% (±0.11) in the casamino acids medium. In comparison, the parental strain showed a 3HV production of 2.9% (±0.5) in the nitrate medium, 3.5% (±0.6) in the ammonium medium, and 0.9% (±0.2) in the casamino acids medium.

In both the OE*phaEC* strain (blue bars) and the parental strain (orange bars), there was a significantly reduced production of 3HB in the medium containing 0.5% casamino acids. For the overexpression strain, no significant differences are observed between production levels in nitrate and ammonium media. In contrast, for the parental strain (HM26), the most substantial production was observed in the ammonium-containing medium (Figure 4).

The results showed that 3HB production only differs significantly between the modified strain and the parental strain in the ammonium-containing medium, where higher synthesis is observed in the parental strain, but in the nitrate-containing medium, a small, non-significant increase in 3HB production is observed in the transformed strain.

Regarding the comparison between media, 3HB levels were considerably lower in the casamino acids medium compared to the nitrate or ammonium media. When comparing the latter two, 3HB levels are higher in the ammonium medium than in the nitrate medium, with these differences being more pronounced in the parental strain.

Concerning 3HV production (Figure 5), a significantly reduced production can also be observed in the medium with casamino acids compared with the media with ammonium and nitrate. Additionally, although there are no significant differences between strains in the production of 3HV, a noticeable increase in 3HV content can be observed in the overexpression strain in nitrate medium compared to the parental strain. 

Table 1 displays the total amount of PHBV quantified with respect to the dried cell weight (% *w*/*w*) in both strains in the three defined media.

Furthermore, the 3HV mol percentage (mol%) was calculated to evaluate the ability of the two strains to incorporate 3HV monomers and to determine whether the overexpression of the *phaEC* genes modified the composition of the PHBV polymer (Table 2). A small increase in the percentage of 3HV was observed in both ammonium and nitrate media in the modified strain, which would result in a polymer with improved physical properties.

### 2.4. Measurement of PHA Synthase Activity 

**Figure 6:**
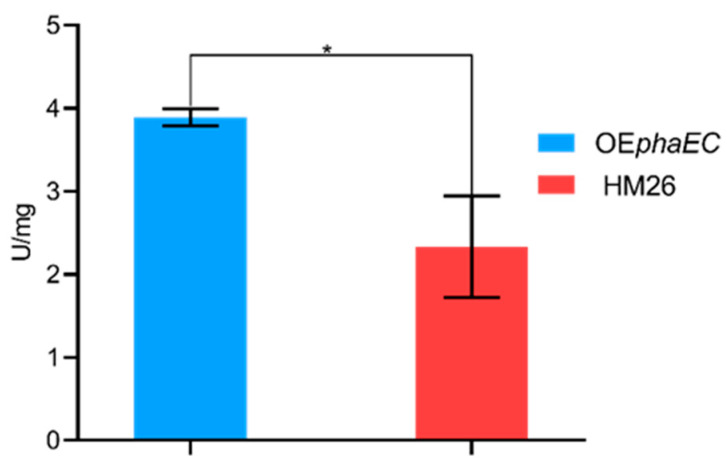
Graphical representation of the mean PHA synthase enzyme activity (U/mg) in both crude protein extracts of the overexpression strain (OE*phaEC*) and the parental strain (Control HM26). *p*-value < 0.05 (*).

Three technical replicas were used to measure the PHA synthase activity from soluble protein extracts obtained from the overexpression and parental strains. As can be observed in Figure 6, the activity is higher in the overexpression strain compared to the control, which confirms the increase in the PHA synthase synthesis. The activities were 3.9 ± 0.1 and 2.3 ± 0.6 (U/mg) in the overexpression and parental strain, respectively.

### 2.5. Protein Extraction of OEphaEC and Parental (HM26) Strains and PhaE Location

**Figure 7:**
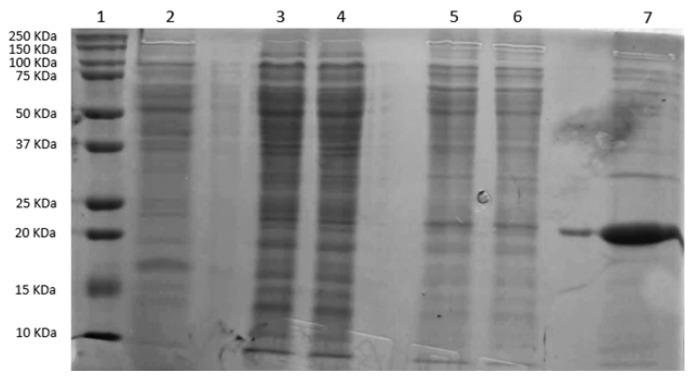
Protein expression analysis by SDS-PAGE. Lane 1: Molecular weight marker (Precision Plus Protein Dual Color, Bio-Rad). Lane 2: Soluble fraction of the HM26 strain. Lanes 3–4: Soluble fraction of the OE*phaEC* strain (two replicates). Lanes 5–6: Insoluble fraction of the OE*phaEC* strain extracted with 0.1% Triton X-100 (two replicates). Lane 7: Insoluble fraction of the *OEphaEC* strain extracted with 1% Triton X-100.

To assess the overexpression of the *phaEC* genes in the OE*phaEC* strain, protein extracts were obtained from both the soluble (buffer A) and insoluble fractions using two concentrations of Triton X-100 (0.1% and 1%; buffer B and C, respectively). The expression profiles of OE*phaEC* and the parental strain HM26 (soluble fraction) are presented in Figure 7. Protein extraction was performed in duplicate for both fractions of the OE*phaEC* strain.

As displayed in Figure 7, a prominent protein band between 20 and 25 kDa was detected in the insoluble fraction of the OE*phaEC* strain extracted with 1% Triton X-100. In contrast, the bands observed in the soluble fraction and in the insoluble fraction extracted with 0.1% Triton X-100 were less intense, suggesting a lower presence of the protein in these fractions. This molecular weight is consistent with the documented size of the PhaE subunit from *H. mediterranei* (20.4 kDa) [42]. A smaller band size is also observed in the soluble fraction, indicating that a certain amount of the PhaE subunit remains soluble under the tested conditions. Additionally, no clear band corresponding to the expected molecular weight of PhaC (54.8 kDa) was observed in any of the fractions analysed from OE*phaEC*.

### 2.6. TEM PHBV Granule Images

In order to observe the PHBV granules with TEM, cells of the overexpression (*OEphaEC*) and parental strain were grown in a medium with 100 mM KNO_3_ and 55.5 mM of glucose. The granules were observed in both exponential and stationary growth phases.

**Figure 8:**
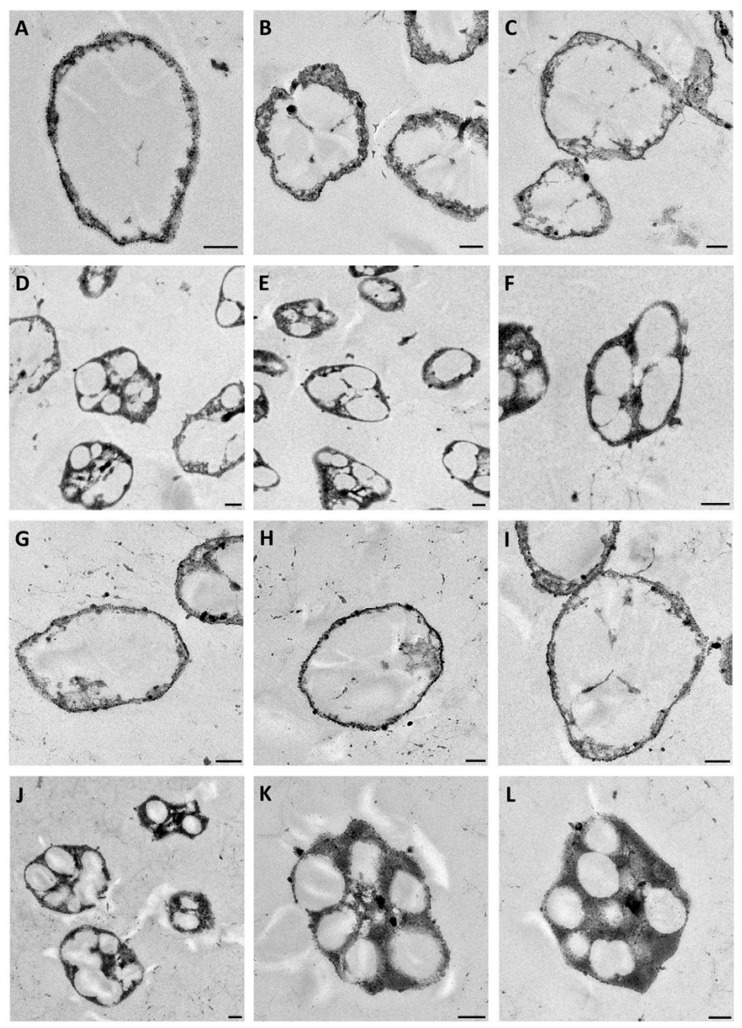
TEM images of the overexpression and parental strains at different growth phases. (**A**–**C**) Overexpression strain at an OD_600_ of 1.8 (exponential phase). (**D**–**F**) Parental strain at an OD_600_ of 2.4 (exponential phase). (**G**–**I**) Overexpression strain at an OD600nm of 3.3 (stationary phase). (**J**–**L**) Parental strain at an OD_600_ of 3.8 (stationary phase). Scale bar: 200 nm.

Figure 8 shows the TEM images of the overexpression strain and parental strain cells at exponential and stationary phases. 

Transmission electron microscopy (TEM) analysis reveals that the OE*phaEC* strain accumulates PHBV in almost the entire cell volume. This biopolymer is stored in a single, large granule occupying most of the cytoplasm. In contrast, TEM images of the parental strain (*H. mediterranei* HM26) indicate a lower accumulation of PHBV, which is distributed among multiple granules of different sizes. No apparent differences in PHBV accumulation are observed between the different growth phases in either strain. In the OE*phaEC* strain, PHBV accumulation is already observed in a single granule occupying almost the entire cytoplasm during the exponential growth phase, with a similar pattern persisting in the stationary phase. Similarly, in the parental strain, PHBV accumulation remains distributed in multiple granules within the cytoplasm across both growth phases. 

Overall, TEM images highlight the higher PHBV accumulation capacity of the OE*phaEC* strain compared to the HM26 strain under identical culture conditions.

## 3. Discussion

In recent years, halophilic microorganisms have attracted much interest due to their biotechnological potential [16,45]. Among them, haloarchaea possess a wide range of metabolic pathways and the ability to synthesise biomolecules of great relevance for biotechnological applications [46]. Notably, species such as *H. mediterranei* produce valuable compounds, like bacterioruberin, a C50 carotenoid with high antioxidant activity and applications in the food, cosmetic, and medical industries [47,48,49]. In addition, *H. mediterranei* plays a crucial role in environmental bioremediation, as it can utilize nitrate (NO_3_^−^) and nitrite (NO_2_^−^) (the least toxic at very low concentrations for most living beings) from the environment either as a nitrogen source for growth or as alternative electron acceptors during denitrification. Thus, a wider spectrum of metabolic capacities makes *H. mediterranei* a promising candidate for wastewater and soil bioremediation [46,50,51,52]. 

In the context of this study, *H. mediterranei* is employed for the optimisation of polyhydroxyalkanoate (PHA) production, specifically poly(3-hydroxybutyrate-co-3-hydroxyvalerate) (PHBV), due to its efficiency and versatility [26,29]. This haloarchaeon is capable of synthesising poly(3-hydroxybutyrate) (PHB), PHBV, and poly(3-hydroxybutyrate-co-3-hydroxy-4-hydroxybutyrate) (PHBV4HB), the latter two exhibiting superior mechanical properties compared to PHB [23]. Furthermore, the genus *Haloferax* has demonstrated particular biotechnological relevance due to its high growth rate and higher PHA production compared to other microorganisms. This genus is not only distinguished by its enhanced production capacity but also by its ability to utilise a wide range of substrates and even wastes as carbon sources for growth and PHA synthesis [23,27,34].

Additionally, *H. mediterranei* is capable of producing over 70% of PHBV in optimal media growth without the addition of any precursors, such as valerate or propionate, which are costly and could be toxic for the microorganisms [34,43,53,54]. PHBV has many advantages over PHB thanks to the incorporation of 3HV monomers, which give better mechanical properties as well as lower brittle and fragility and present more flexibility and resistance. The optimization of culture media has been one of the most extensively studied strategies to enhance the competitiveness of PHA production, particularly PHBV, given that the primary challenge is its high production cost compared to conventional plastics. Several studies have shown significant progress in this area, from the incorporation of industrial waste as an alternative carbon source to the modification of C/N ratios [22,30,32,55,56,57].

Beyond media optimisation, another promising and complementary strategy to reduce production costs lies in the development of PHA overproducing strains capable of maximizing yield [43]. In this context, the present study addresses an innovative approach: the construction and characterisation of an *H. mediterranei* strain that overexpresses the *phaE* and *phaC* genes, which encode PHA synthase, the central enzyme in the biosynthesis of these biopolymers. To the best of our knowledge, this is the first time that homologous overexpression has been used as a strategy to produce PHBV by a haloarchaeon. To evaluate the efficiency of this engineered strain, it is essential to analyse its growth under varying culture conditions and assess its PHBV production capability in comparison to the parental strain, *H. mediterranei* HM26.

The production of PHBV was studied in defined media with different nitrogen sources. The medium with 100 mM potassium nitrate was used, as it ensures optimal growth of *H. mediterranei,* and previous work by our group showed optimal results for PHBV production under these conditions [30]. To test nitrogen-restricted conditions, *H. mediterranei* was grown with 10 mM ammonium as the nitrogen source, as this is the preferred source for nitrogen assimilation. Under these conditions, the C/N ratio is 28.6. On the other hand, growth in the presence of casamino acids as a nitrogen source was also studied to test the effect of an amino acid acid-rich medium on the composition of the synthesised PHBV.

Regarding the growth rates of both the OE*phaEC* and parental strain, the results show that cells grew worse in media with nitrate as the nitrogen source. As previously described, *H. mediterranei* did not synthesise the enzymes of the nitrate assimilation pathway unless ammonium was depleted from the culture media, indicating that ammonium was the preferred nitrogen source [58]. Unlike ammonium assimilation, nitrate assimilation requires two additional reactions. First, assimilatory nitrate reductase (Nas) converts nitrate to nitrite, which is then converted to ammonium by nitrite reductase (NiR) so it can be incorporated into biomolecules, which constitutes a higher energetic expenditure for the cell [59]. Therefore, the results obtained in this study regarding growth rates are consistent with the higher energy demand required to assimilate nitrate as the sole nitrogen source.

Jantzer et al. [60], in an effort to determine the optimal growth conditions for *Haloferax volcanii*, tested two carbon sources, glucose and casamino acids, at various concentrations. Cultures with casamino acids showed a linear trend with respect to the tested concentrations (up to 0.5% casamino acids), achieving higher OD_600_ values compared to those with glucose as the carbon source. In a similar study, Esclapez et al. [58] reported that the growth rate of *H. mediterranei* in complex media supplemented with 0.5% casamino acids (as a nitrogen source) was higher than in media with other nitrogen sources, such as ammonium and nitrate. Although the present study investigates casamino acids as the nitrogen source, it is reasonable to observe a higher growth rate compared with nitrate medium, as casamino acids also serve as a carbon source, allowing *H. mediterrane*i to grow rapidly. Notably, when comparing the duration of the lag phase, the cultures with casamino acids showed a lag phase of approximately 10 h, whereas, in the other media, the lag phase was longer (around 17 h and 41 h for ammonium and nitrate media, respectively). These results indicate that the medium containing 0.5% casamino acids and the medium with 10 mM of ammonium are better for *H. mediterranei* growth compared to the medium containing nitrate as a nitrogen source. Interestingly, although the medium containing 1% glucose and 0.5% casamino acids was identified as optimal for growth, it proved to be less efficient for PHBV production, despite the abundant carbon source. In this study, a significantly lower production of 3HB and 3HV monomers was observed in the medium supplemented with casamino acids for both the OE*phaEC* and parental (HM26) strains (Figure 4 and Figure 5). However, this medium favoured the incorporation of 3HV monomers into the polymer, as evidenced by a higher mol% of 3HV compared to the medium with ammonium as the nitrogen source in both strains (Table 2). This is consistent with the fact that *H. mediterrane*i can synthesise propionyl-CoA, the precursor of 3HV, from amino acids [34]. The synthesis of a polymer with approximately 20 mol% of 3HV was reported when *H. mediterranei* was cultivated in an amino acid-rich medium [61].

But, upon examining the mol% of 3HV, the results indicated that the highest incorporation of 3HV in the OE*phaEC* strain occurs in the medium with nitrate as the nitrogen source compared to the other media tested. In contrast, the parental strain showed a higher incorporation of 3HV in the medium containing casamino acids. However, the OE*phaEC* strain generally demonstrated a tendency for higher 3HV incorporation, particularly in both the nitrate and ammonium-based media (Table 2). This suggests that the increased availability of PHA synthase in the OE*phaEC* strain likely facilitates the enhanced incorporation of 3HV. Moreover, a 40% increase in 3HV content was observed in the OE*phaEC* strain in the nitrate medium relative to the parental strain under the same conditions (Figure 5). This fact suggests that the nitrate medium may favour the incorporation of 3HV in the modified strain.

Furthermore, the OE*phaEC* strain demonstrated higher specific activity of the PHA synthase compared to the parental strain, which could be attributed to the greater availability of active synthase in the engineered strain (Figure 6). As shown in Figure 7, the PhaE subunit of the PHA synthase is present in all three protein fractions obtained from the OE*phaEC* strain: the soluble fraction and those extracted with 0.1% and 1% Triton X-100. However, a more intense band is observed in the insoluble fraction extracted with 1% Triton X-100, where PHBV granules are retained. Lu et al. [42] demonstrated that both subunits of the PHA synthase from *H. mediterranei* are essential for enzymatic activity (1:1 ratio). They confirmed the presence of both subunits in the soluble fraction as well as in PHBV granules. Based on these findings, they suggested that both subunits are stably associated with PHBV granules. Our results are consistent with these findings, as we detected the PhaE subunit in both crude cell extracts and insoluble fractions, where the granules are retained in our study. Additionally, we confirm a higher presence of PhaE in the insoluble fraction, suggesting a strong association with the granules. This is explained because the homologous overexpression of the *phaEC* genes results in an increased amount of the PhaE subunit, which appears to remain more tightly bound to the granules. However, no clear band of the overexpression of the PhaC subunit could be detected in either the soluble or the insoluble fraction. It is possible that this subunit is distributed in both fractions since the activity is detected in the soluble fraction and the increased enzymatic activity detected in the OE*phaEC* strain suggests a higher presence of this subunit compared to the crude extract of the control strain. Moreover, previous studies have determined the instability of the PhaC subunit when overexpressed and that it is precipitated in the form of aggregates [62,63]. Similar behaviour could be occurring in *H. mediterranei* that could lead to the degradation of the enzyme in the stationary phase.

PhaC in *H. mediterranei* possesses a longer C-terminal region compared to bacterial PhaC homologs. This extended C-terminal domain may play an essential role in the correct synthesis of PHBV. The AlphaFold structural model of PhaC (AF-I3R9Z4-F1-v4) (https://alphafold.ebi.ac.uk/ accessed on 11 March 2025) [64] indicates that this C-terminal region is a disordered region with a helix–hairpin–helix DNA-binding domain whose function is not known. This domain, absent in PhaC from bacteria, has been described in a new type of phasin, PhaH, in *Caulobacter crescentus*, and its function has been related to the size of the PHB granules and their distribution in the cell, as it has been proposed to allow a better interaction of the granules with the nucleoid, allowing the carbonosomes to more evenly distribute inside the cells [65]. However, despite advances in understanding the PHBV synthesis pathway in *H. mediterranei,* the precise role of the PhaE subunit remains unclear [9,66]. Further studies are needed to elucidate its function and the role of the extended C-terminal domain of PhaC in PHBV biosynthesis.

The literature has documented that several factors influence the molecular weight of the accumulated PHBV, with the amount of PHA synthase being one of them. Several studies have reported an inverse relationship between the quantity of this enzyme and the molecular weight of the polymer, suggesting that an increased level of synthase may promote the production of polymers with lower molecular weight [61,67,68,69]. However, it is important to emphasize that a higher synthase activity is associated with higher polymer accumulation. For instance, in *E. coli* expressing the *phaABC* operon from *Ralstonia eutropha*, a positive correlation has been observed between PhaC enzymatic activity and PHB accumulation [70]. In our study, the molecular weight of the produced PHBV was not determined. Nevertheless, if a reduction in molecular weight were to occur, it could provide advantages in terms of polymer extraction. It is well established that the molecular weight of PHBV is a key factor determining its solubility during solvent-based extraction, which is the most commonly employed method for PHA recovery [71,72]. Consequently, polymers with slightly lower molecular weight tend to exhibit greater solubility, thereby facilitating the extraction process. Thus, a potential decrease in molecular weight could be beneficial for improving the efficiency of PHBV recovery.

Moreover, based on the TEM images of cells grown in defined media with nitrate, we observed that the overexpressing strain exhibited a single, large granule that occupied nearly the entire cytoplasm. In contrast, the parental strain accumulated PHBV in multiple smaller granules, without occupying the whole cytoplasm. These images show that a higher amount of PHBV accumulated in the OE*phaEC* strain compared to the parental strain. These results are consistent with the monomer analysis by GC-MS, which showed a 20% increase in PHBV content in the OE*phaEC* strain compared to the parental strain in nitrate medium, which is the same medium used for the TEM imaging. One of the main advantages of this observation is that the higher cellular PHA content could facilitate the subsequent recovery of the polymer, as fewer solvents or chemical/enzymatic digestive agents would be required for extraction [43,73].

Optimising cultivation conditions or engineering overproducing strains of PHBV is crucial to ensure that this polymer can be competitively applied across various fields. In this study, the chosen strategy involved the homologous overexpression of the *phaEC* genes, which encode the PHA synthase of *H. mediterranei*, resulting in the OE*phaEC* strain. The most remarkable finding was the significantly enhanced ability of this strain to incorporate 3HV into the monomer, a key factor in improving the versatility of PHBV for diverse applications, particularly in the medical field [6,74,75,76]. Similarly, other strategies have successfully improved the cellular PHA content in *H. mediterranei.* For example, Lin et al. [77] managed to repress genes encoding citrate synthase (*citZ* and *gltA*), achieving a 13.4% increase in cellular PHBV content (from 48.92 to 55.49% of PHBV content). Furthermore, the same research group deleted genes related to exopolysaccharide (EPS) synthesis in this haloarchaeon (ΔEPS strain), resulting in a strain capable of accumulating 8% more PHBV compared to the wild-type strain (from a content of PHBV of 44.5 to 48%) [78]. In the present study, in the 100 mM nitrate medium, the OE*phaEC* strain exhibited a 20% increase in PHBV content (the sum of 3HB and 3HV) compared to the parental strain. Moreover, the 3HV content increased by 40%, a significant improvement in polymer composition. This observation aligns with findings in *E. coli,* where increasing the copy number of the pha*CAB* genes boosted PHB productivity from 0.1 g/L to 1.3 g/L, showing that higher expression levels of PHA synthesis genes lead to increased polymer production [79]. Additionally, Yang et al. [80] demonstrated that the deletion of the three origins of replication *in H. mediterranei* led to a 13.7% increase in PHBV content compared to ΔEPS strain cultivated in MS medium, which is a favourable medium to synthesize PHBV (from 47.06 to a content of 53.49%). This improvement was attributed to an increase in the copy number of the plasmid pHM300, where the *phaEC* genes were overexpressed. These findings underscore that constitutive expression systems for PHA synthesis-related genes in *H. mediterranei* represent an effective strategy to enhance PHBV production, particularly regarding the incorporation of 3HV into the polymer. However, the genetic stability of the OE*phaEC* strain requires further monitoring, as plasmid-based expression systems may lead to instability [77], especially in defined media, such as those lacking uracil. Despite this limitation, plasmid-based expression remains a widely used approach in both bacteria and archaea to enhance PHA production. Notably, a similar strategy has been employed in *R. eutropha*, where the overexpression of the *phbC* gene, encoding the PhaC synthase, resulted in a 40.5% increase in PHB content compared to the parental strain (from 5.51% of PHB to 7.74%) [81].

## 4. Materials and Methods

### 4.1. PHA Synthase Overexpression Plasmid Construction

The overexpression strain HM26-OE*phaEC* was constructed using the pTA-1992 plasmid, kindly provided by Dr. Thorsten Allers (Faculty of Medicine and Health Sciences, University of Nottingham). First, the *phaE* (E6P09_RS17990) and *phaC* (E6P09_RS17995) genes encoding PHA synthase in *H. mediterranei,* located in the megaplasmid pHME332, were amplified in a single amplicon by PCR using specific primers with restriction sites for *PciI* and *BamHI* for insertion into the pTA-1992 plasmid (forward *phaE*: 5′-CCGAGAGAGTAcATGtCCATGTCACAAC-3′ and reverse *phaC*: 5′-GAGTTCCAGGatCCACACACATCGG-3′). With these primers, a product of 2138 bp was obtained and purified from agarose gel with the *GFX PCR DNA and Gel Band Purification Kit* from Cytiva.

The pTA-1992 plasmid contains the highly active synthetic promoter p.*syn* that allows the constitutive overexpression of proteins tagged with a histidine tail at the N-terminus. The vector contains two markers that allow selection in the absence of uracil and/or thymidine, *pyrE2* and *hdrB* [82]. Both vector and product were then subjected to the restriction reaction with the specified enzymes, and the resulting products were purified using the *GFX PCR DNA* and *Gel Band Purification Kit* (Cytiva) and were ligated using T4 DNA ligase at an insert/vector ratio of 2. 

The recombinant plasmid was first transformed into *E. coli* DH5α by the heat shock method, and ampicillin-resistant cells were selected. The plasmid was extracted using E.Z.N.A.^®^ Plasmid DNA Mini Kit I (Omega bio-tek, Norcross, GA, USA), and the construction was checked by PCR. *E. coli* JM110 was then transformed with the plasmid using the same method as described above in order to obtain a plasmid without methylation patterns. Ampicillin-resistant cells were selected, and the plasmid was isolated using E.Z.N.A.^®^ Plasmid DNA Mini Kit I. The correct construction of the recombinant plasmid was verified by PCR using vector-specific primers (forward: 5′-TCTCGGCGGTTCGAGAATCGA-3′, reverse: 5′-GATGGTCCAGAGGTGCGGC-3′) and Sanger sequencing. After verification of the recombinant plasmid construct, *H*. *mediterranei* HM26 [83], a strain auxotrophic for uracil, was transformed using the PEG-mediated method established for haloarchaea in previous studies [84]. Cells subjected to the transformation process are selected in defined media lacking uracil. Different colonies are checked by plasmid extraction, fragment amplification by PCR, and Sanger sequencing using the pTA1992 primers. All PCRs performed in this work were carried out using Phusion™ High-Fidelity DNA Polymerase (Thermo Fisher Scientific, Waltham, MA, USA). 

### 4.2. Culture Growth Conditions

*H. mediterranei* HM26-OE*phaEC* and HM26 (parental strain) were used for all the experiments. For growth characterization, three defined media were prepared in which the nitrogen source was different: 100 mM potassium nitrate (KNO_3_) (1% *w*/*v*), 10 mM ammonium chloride (NH_4_Cl), and 0.5% of casamino acids (cas). All media contain salts (seawater, SW) at a final concentration of 25% with the following composition: 3.34 M NaCl, 0.2 M MgSO_4_·7H_2_O, 0.17 M MgCl_2_·6H_2_O, 66.67 mM KCl, 2 mM NaHCO_3_, 5.67 mM NaBr, and 8.25 mM CaCl_2_ [85]. Cultures were buffered with 100 mM (3-(N-morpholino) propanesulfonic acid) (MOPS). pH was adjusted to 7.3 with NaOH. After autoclaving, all cultures were supplemented with 1 mM of inorganic phosphate (Na_2_HPO_4_/NaH_2_PO), 55.5 mM of glucose, and 0.030 mM FeCl_3_. The defined media for the parental strain were also supplemented with 0.45 mM of uracil. All cultures were grown in a 0.5 L Erlenmeyer flask at 42 °C with shaking (170 rpm) and maintaining an air-to-culture volume ratio of 3:1.

The cultures were inoculated with preadapted inoculum (previously grown under the same conditions) to an initial optical density at 600 nm (OD_600_) of 0.02 and were incubated until the stationary phase was reached.

Cellular growth was monitored spectrophotometrically by measuring the OD_600_ using a Cary60 UV-Vis spectrophotometer (Agilent, Santa Clara, CA, USA). To calculate the specific growth rates (μ), growth curves were plotted on a semi-logarithmic scale (ln OD_600_ vs. time) for each condition and each culture replicate. Slopes in the exponential growth phase were calculated, and specific growth rates in each condition were calculated as the average of the slopes of the three replicates. 

### 4.3. PHBV Quantification from H. mediterranei HM26-OEphaEC and the Parental Strain HM26

GC-MS analysis was performed for the identification and quantification of the synthesised PHBV (mg of 3HB or 3HV/ mg cell dry weight (CDW)). First, cells from 333 mL cultures grown in a 1 L Erlenmeyer flask were harvested from stationary phase cultures by centrifugation at 16,249× *g* for 30 min. The pellets were then washed twice with 10% (*w*/*v*) of NaCl and centrifuged again. Pellets were frozen at −80 °C and lyophilized. Three biological replicates of each culture condition and strain were analysed, and three technical replicates of each biological replicate were subjected to methanolysis. Briefly, 50 mg of lyophilised cells were mixed with a solution containing 2 mL of chloroform, 0.3 mL of 98% sulfuric acid, 1.7 mL of methanol, and 10 mg of benzoic acid in 1 mL of methanol, which was used as the internal standard. The reaction was kept at 100 °C for 140 min. After cooling, 1 mL of distilled water was added, resulting in the formation of two phases. The lower chloroform layer was used for GC-MS analysis as described previously [7]. Pure PHBV (Cymit Quimica, CAS: 80181-31-3) was used as a standard in a range from 5 to 50 mg. The analysis was performed on a GC/MS system (Agilent Technologies 7890A) coupled with a mass detector operated in SCAN mode. The column used was a 30 m × 250 µm × 0.25 µm HP-5MS (Agilent). The injection was performed with a split ratio of 1:100 and a total volume of 1 µL. The oven temperature was initially set to 40 °C for 2 min and ramped from 40 °C to 280 °C at a rate of 10 °C/min, holding 280 °C for 5 min. The total flow rate was 104 mL/min with helium as the carrier gas. 

### 4.4. PHA Synthase Activity Assay of OEphaEC and Parental (HM26) Strains

The enzyme activity was recorded spectrophotometrically at 412 nm, recording the release of CoA during the polymerisation of the substrate 3-hydroxybutyryl-CoA by the PHA synthase, as described previously by Lu et al. [42]. A total of 500 µg of the soluble fraction, from the overexpression and the parental strains, grown in the same medium used for protein extraction (see next method) was used. All the assays were performed in a final volume of 1 mL at 38 °C. The composition of the reaction buffer was 20 mM Tris-HCl pH 7.5, 3.4 M KCl, and 100 µM of Mg(CH_3_COO)_2_. Additionally, 1 mM de 5,5′-dithiobis-(2-nitrobenzoic acid), 1 g/L bovine serum albumin, and 100 µM of 3-hydroxybutyryl-CoA were added in all the assays.

All the compounds were incubated for 2 min at 38 °C, except the substrate. The reaction was initiated by the addition of 10 µg of 3-hydroxybutyryl-CoA. Change in the absorbance was recorded every 10 s over a 2 min period. The calculation of PHA synthase activity was based on the Lambert–Beer law: ΔA/min = ε × l × (ΔC/min). The molar extinction coefficient of CoA used was 13,600 M^−1^ cm^−1^. One unit of enzyme activity was defined as the amount of enzyme that catalyzes the generation of 1 nmol of CoA per minute. The specific activity was calculated by expressing the units of enzyme activity per mg of protein. 

### 4.5. Protein Extraction of the OEphaEC and Parental (HM26) Strains

Three OE*phaEC* cultures (500 mL each) were grown in 3 L flasks containing 20% SW, 15 mM NH_4_Cl, 0.1 MOPS, 1 mM Na_2_HPO_4_/NaH_2_PO_4_, 0.5% glucose, and 0.005 g/L FeCl_3_ until the stationary phase. The parental strain was cultured in the same medium supplemented with uracil at a final concentration of 0.05 g/L in a 250 mL flask. The air-to-liquid ratio was maintained at 1:6. Cultures were inoculated at 0.5% of the total volume and incubated at 42 °C with agitation for 48 h.

Proteins were extracted from both the soluble and insoluble fractions. For the insoluble fraction, two Triton X-100 concentrations were used: 0.1% and 1% (*v*/*v*). The extraction buffers used were buffer A (soluble fraction): 20 mM Tris-HCl, 1.5 M NaCl, pH 7.2, and 1 mM phenylmethylsulfonyl fluoride (PMSF); buffer B: 20 mM Tris-HCl, 1.5 M NaCl, 0.1% Triton X-100, and pH 7.2; and buffer C: 20 mM Tris-HCl, 1.5 M NaCl, 1% Triton X-100, and pH 7.2.

Biomass was harvested by centrifugation at 16,249× *g* for 30 min at 4 °C. The soluble fraction was obtained by resuspending the pellet at 30% in buffer A. Cell lysis was performed by sonication at 30% amplitude, applying 10 s pulses (on) and 35 s intervals (off) for a total of 8 min. The lysate was centrifuged at 31,152× *g* for 30 min at 4 °C. The supernatant was collected as the soluble protein fraction, while the pellet was resuspended at 10% in buffer B and incubated with agitation at 4 °C for 24 h. The suspension was centrifuged under the same conditions, and the supernatant was collected as the fraction extracted with 0.1% Triton X-100. The remaining pellet was resuspended at 10% in buffer C and incubated in the same conditions. After centrifugation at 31,152× *g* for 30 min at 4 °C, the supernatant was collected as the fraction extracted with 1% Triton X-100.

Proteins from each fraction were precipitated using 20% trichloroacetic acid (TCA) and subjected to three washing steps with a 1:1 acetone–MQ water solution. The precipitated proteins were analysed by sodium dodecyl sulfate–polyacrylamide gel electrophoresis (SDS-PAGE) under denaturing conditions.

### 4.6. TEM Analysis 

*Modified OEphaEC and control HM26* strains were grown in defined media containing 100 mM potassium nitrate to the stationary phase. PHA granule formation was analysed in both exponential and stationary phases. Briefly, 10 mL of the cell culture in both phases (OD_600_ of 1.8 and 3.3 for OE*phaEC* and 2.4 and 3.8 for HM26) was harvested by centrifugation at 7197× *g,* and the pellet was washed once with 10% NaCl. Subsequently, the cells were fixed in a solution containing 2.5% glutaraldehyde (Electron Microscopy Sciences, Hatfield, PA, USA) in PBS (10% NaCl, 0.1 M sodium phosphate buffer, pH 7.2). After at least 2 h of fixation at room temperature, the samples were rinsed with PBS, and the pellets were embedded in small blocks of 2% agar (Sigma-Aldrich, St. Louis, MO, USA) measuring approximately 5 × 5 × 1 mm. Post-fixation was then performed using 1% osmium tetroxide. Following fixation, the cells were dehydrated through a graded series of ethanol concentrations (70%, 95%, and 100%), immersed in propylene oxide (Electron Microscopy Sciences, Hatfield, PA, USA) for solvent substitution, and finally embedded in EPON-812 epoxy resin (Electron Microscopy Sciences, Hatfield, PA, USA). Ultrathin sections were prepared and double stained with 5% uranyl acetate and 2.5% lead citrate. Finally, the specimens were examined using a JEOL JEM-1400 Plus transmission electron microscope equipped with a Gatan Orius digital camera (Gatan, Pleasanton, CA, USA) for image acquisition.

### 4.7. Statistical Analysis

All experiments were carried out in triplicate. For all the results, GraphPad Prism version 8.0.2 was used. For the graphical representation of the kinetic growth of both *H. mediterranei* strains, the mean of each point was calculated with the standard deviation (SD). For PHBV quantification, 3 technical replicas of each biological replica were used to perform methanolysis, and the results were expressed as the mean ± SD. For the growth rate and quantification of PHBV, differences within and between groups were evaluated by one-way ANOVA followed by a multicomparison Tukey’s test. To assess PHA synthase activity, Student’s *t*-test was performed. In all the analyses, a value of *p* < 0.05 was considered significant.

## 5. Conclusions

In this work, we have, for the first time, achieved homologous overexpression of the *phaEC* genes of *H. mediterranei* through a constitutive expression system. This strategy successfully enhanced PHBV production in a defined medium with nitrate as the nitrogen source, with a considerable improvement in the incorporation of 3HV into the polymer in nitrate and ammonium media. This increase alone would be beneficial for the production of PHBV on an industrial level, although its complementation with other strategies, such as EPS deletion, could lead to even higher yields that are necessary for industrial applications. The regulatory role of both PhaE and PhaC subunits in *H. mediterranei* should be explored in depth to understand the mechanism that differentiates haloarchaea in the process of the regulated synthesis and segregation of PHBV granules.

## Figures and Tables

**Table 1:** Total % of PHBV (*w*/*w*) of the OE*phaEC* and HM26 strain in different defined media.

Media	%PHBV (OE*phaEC*)	%PHBV (HM26)
KNO_3_	32.9 ± 1.3	27.4 ± 3.2
NH_4_Cl	34.3 ± 4.8	44.2 ± 1.41
Casamino acids	5.2 ± 0.8	8.1 ± 3.1

**Table 2:** Values of the mol% of 3HV in the PHBV polymer obtained in the different culture media with the overexpression strain (*OEphaEC*) and parental strain (HM26).

Media	%PHBV (OE*phaEC*)	%PHBV (HM26)
KNO_3_	10.8 ± 0.3	9.2 ± 0.4
NH_4_Cl	8.4 ± 0.7	7.0 ± 1.0
Casamino acids	10.2 ± 0.5	11 ± 4

## Data Availability

The raw data supporting the conclusions of this article will be made available by the authors on request.
